# Two Letters on Animal Electricity

**Published:** 1792

**Authors:** Eusebius Valli

**Affiliations:** of the University of Pisa.


XIX. *Two Letters on Animal Electricity.* *By* Eusebius Valli, *M. D. of the University of Pisa.*—Vide *Journal de Physique.* 4to. Paris, 1792.

THE facts relative to animal electricity, described in the preceding article, appeared to Dr. Valli of so much importance, that he was induced to repeat the experiments of Professor Galvani, and to institute many new ones on the same subject, which serve considerably to illustrate trate and extend the difcovery in queftion. The following account of his refearches cannot fail, therefore, of being highly acceptable to our readers; and we give it to them with the more fatisfaction, as the candid and ingenious author, who is at prefent in London, has more than once gratified us with a repetition of many of his experiments:

EXPERIMENT I.

The author opened the belly of a frog, fo as to lay bare the fpine, and difcover the crural nerves that iffue from it. Two lines above their infertion he cut the frog in two, and, paffing his fciffars under the origin of thofe nerves, removed the remainder of the vertebral column, leaving only the vertebra that united the bundle of nerves. He furrounded this vertebra with a piece of thin lead, which formed a coating, and he removed the integuments of the lower part of the frog, fo as to lay bare the mufcles. Having thus prepared it, he touched at the fame time with an iron wire, difpofed as a conductor, and infulated (by means of a piece of fealing wax, which ferved as a handle to it) the leaden coating and the mufcles of the frog, and he obferved all the phenomena phenomena defcribed by Profeffor Galvani.— Thefe phenomena, he tells us, take place equally whether the animal be infulated or not. He employed conductors of different metals, having obferved that by fo doing all the electrical phenomena become more apparent. Thofe of filver feemed to him to be the beft.

### Experiment II.

Two frogs, prepared in the manner above defcribed, after having ceafed to give any figns of life, experienced a very great tremor when touched by the conductor.

### Experiment III.

While he made thefe experiments on one frog, he left another at reft that he had prepared at the fame time. When the firft had ceafed to move, and was entirely extinct, he took the fecond, which in an hour and a half had loft none of the faculties that he had exhaufted in the firft, and, notwithftanding this delay, he made on this the fame experiments as with the other, and obtained the fame refults.

### Experiment IV.

He had a frog, the crural nerve of which, and the extremity of the body correfponding with it, fhewed no figns of feeling. On inquiring into the caufe of this, he found that the filaments of the nerve were ruptured. Having brought them together, he coated them at the point of reunion, and, on applying the conductor, a tremor was excited in the leg. When this motion had ceafed he divided the oppofite nerve, and after collecting the filaments, and placing them at a diftance from the limb, touched it feveral times with the conductor, but without exciting any movement.

### Experiment V.

He prepared two other frogs, taking care to feparate the nervous filaments of each crural nerve. In making the experiment they were as much agitated as thofe whofe nerves remained in their natural pofition.

### Experiment VI.

After having fatigued for an hour and a half two frogs, prepared as ufual, he left them at reft an hour and ten minutes. He then attempted to excite motion with a conductor made of copper covered with silver; one of them sprang from the plate of glass on which it was placed, and remained afterwards twenty minutes affording only slight tremors. The first movement in the other was less violent, but it was nevertheless much agitated, and continued to be so full as long as the other.

### Experiment VII.

Wishing to ascertain how long frogs are capable of supporting this state, he prepared two at ten o'clock at night. At seven the next morning he found them feeble, but not without motion. Both of them, on being subjected to the usual experiment, afforded a slight tremulation. An hour afterwards they ceased to give signs of electricity to any of the means that were employed.

### Experiment VIII.

At other times he has left, in the same manner, during the night, frogs prepared as usual, but in the morning has found them dry, and yielding no sign of electricity.

### Experiment IX.

After feparating fome mufcles from the body of a frog, and lacerating them, it was not poffible to excite their irritability by a mechanical ftimulus, but the conductor excited it.—" Does " the motion of the mufcles produced by the " irritation thus excited, or by the nerves that " are diftributed to them," afks our author, " differ from that which refults from the dif- " charge of electric matter? And which of " thefe motions approaches the neareft to the " voluntary motions?"

### Experiment X.

The brain of a frog having been laid bare, and irritated, it died apparently in convulfions; but on applying the conductor the mufcles of the animal immediately contracted.

### Experiment XI.

The preceding experiment, our author tells us, was repeated in order to compare it with what would happen to frogs dying without convulfions; comparifon, he remarks, being the rule we ought to follow when we have no other that is better or more accurate. No difference was perceptible; and therefore he concludes that the animal loſt nothing in the convulſions, and that the principle of life was preſerved. But a man, he obſerves, agitated by convulſions and nervous affections is exceſſively debilitated. This leads him to offer a query, which he thinks we ſhall ſome day be able to ſolve — "Does "there exiſt in the animal œconomy another "agent beſides electricity?"

### Experiment XII.

He applied opium to one of the crural nerves. The limb ſeemed to ſuffer by it a little, as did alſo the other limb; but after ſome time both recovered their former vigour.

### Experiment XIII.

Opium applied to the extremity of a divided nerve produced no apparent diminution of electricity in the part.

### Experiment XIV.

After having kept for ten minutes one of the extremities of a prepared frog in a tepid bath of opium, the limb, on being ſubjected to the uſual experiments, became fatigued in leſs than a quara quarter of an hour, and when it ſeemed to have loſt its electricity he proceeded to the other limb, which moved vigorouſly on applying the conductor, and gave ſigns of electricity for at leaſt an hour and a half.

### Experiment XV.

Three frogs were made to ſwallow a ſolution of opium in warm water; and at the end of an hour were prepared, and placed in a ſolution of opium; notwithſtanding which they ſtill continued to move when excited.

### Experiment XVI.

After bathing with a ſimilar ſolution the adductor and triceps muſcles of the thigh of a frog, their motions ſeemed to be ſtronger than uſual; but the author thinks that this might be accidental.

### Experiment XVII.

A ſolution of opium was poured between the ſkin and the muſcles of the thighs of two frogs, but without impairing their electricity.

### Experiment XVIII.

A ſolution of opium was introduced between the fibres of the triceps muſcle of a frog, the extremities of which were already impregnated with the ſame ſolution. This frog remained immoveable.

### Experiment XIX.

In ſix other frogs the phenomena were different, the opium having neither weakened nor ſuſpended their electricity.

### Experiment XX.

Opium applied to the inſulated muſcles of frogs extinguiſhed their electricity only in one inſtance out of twenty, but in that one the effect, we are told, was inſtantaneous. Theſe facts, the author obſerves, embarraſs him.

### Experiment XXI.

The muſcles of living frogs ceaſed to be excited by mechanical ſtimuli after opium had been applied to them, or to their nerves, and yet were excited by the conductor as often as it was employed.

### Experiment XXII.

The author laid bare the brains of four frogs, and applied opium to them. They fell down as if ſtruck with lightning. He prepared them for the experiments, leaving the lower extremities united to the trunk and the head. The ſpine was cut off, and ſeparated below the crural nerves. Upon coating them, and applying the conductor, the uſual phenomena enſued.

### Experiment XXIII.

Inſtead of opium, other extraneous ſubſtances were applied to the brains of different frogs, but without ſeeming in the leaſt to impair their electricity.

### Experiment XXIV.

Six frogs were made to ſwallow a conſiderable quantity of opium; but in none of them was the electric principle apparently weakened by it.

### Experiment XXV.

Tobacco, in powder, rendered five frogs profoundly ſtupid and inſenſible; nevertheleſs they gave the uſual ſigns of electricity with the conductor.

### Experiment XXVI.

The author coated the nerves of the legs of lizards, and obtained ſlight contractions; but on coating the marrow of the tail the motions he excited were more violent, and laſted longer.

### Experiment XXVII.

Lizards that had been poiſoned with tobacco, and died in convulſions, were found not to have loſt their electricity. In numerous experiments to aſcertain this fact, not one, we are told, was contrary to it.

### Experiment XXVIII.

The author coated, near the head, the ſpinal marrow of two tench, each of which weighed about an ounce and a half. They raiſed their fins five or ſix times when excited, but in leſs than two minutes ceaſed to yield any motion.

### Experiment XXIX.

An eel was cut in two, and the ſpinal marrow of each of the two portions was coated with tin foil. On applying the conductor the tail ſtruck violently, as if it had been in water; and on continuing to touch it it turned different ways; but infenfibly grew weaker, and in lefs than three quarters of an hour its electricity was extinct.

This principle was lefs ftrong in the part next the head, but it lafted about five minutes longer.

### Experiment XXX.

The wing of a lark, prepared in the ufual way by laying bare the mufcles, experienced flight movements for three minutes, but the legs could not be excited. The author afcribes this failure to the fmallnefs of the crural nerves in this bird.

### Experiment XXXI.

A kitten, juft born, was carefully prepared, and contractions were excited in it for feven or eight minutes; but no movement could be produced in the mufcles of the tongue or larynx.

### Experiment XXXII.

The author prepared two dogs. The firft, from a want of proper precaution in the experiment, afforded nothing; but the fecond, which he killed by a blow of the head, yielded ftrong motions, and in particular one of its fore paws bent bent five or ſix times, as in walking. The hyogloſſi and geniogloſſi muſcles trembled ſeveral times. Thoſe of the larynx alſo, the nerves of which had been coated, were affected in the ſame manner.

The heart did not palpitate, although the eighth pair of nerves were coated while that viſcus was reeking and warm. In an hour all was over.

We now come to the author's ſecond letter.

From ſome of his firſt experiments he had been led to aſſert that a ligature paſſed round a nerve prevents the paſſage of the electric fluid; but one of his friends (Mr. Fattori) having informed him that this is not always true, he repeated the experiment, and found that by making the ligature on the nerve cloſe to its inſertion into the muſcle the motion is entirely prevented; but that, on the contrary, if the ligature be at a diſtance from the muſcle, the experiment ſucceeds very well.

There is no part of an animal, he obſerves, that is not a conductor of electricity, and he is unable to ſay which is the beſt, becauſe he has ſeen an infinite number of anomalous appearances.

The

The ſhocks, he aſſures us, are, in general, ſtronger if the conductor is carried from the muſcles to the coated nerve, inſtead of its being carried from the latter to the muſcles.—If this laſt method is adopted, when the electricity is ſo weak that it is nearly extinct, no motion enſues; yet even then it may be excited by the other proceſs. This fact, as the author obſerves, is ſingular, and merits the attention of philoſophers.

Slight wounds of the brain in frogs, ſometimes, we are told, occaſion convulſions or palſy, and at other times produce no ill effect.

When the wound of the brain is more conſiderable, the animal ſometimes dies ſuddenly; but the author has ſeen frogs ſurvive for ſeveral days the deſtruction or laceration of this organ.

In general, however, he has found that frogs whoſe brain has been lacerated have yielded electric movements only for about two minutes. He ſuſpected that this might be owing not to the want of electricity, but to the nerve becoming a non-conductor, or to ſome change in the ſtate of the muſcular fibre: and having in one inſtance obtained movements by ſubſtituting, in the place of the nerve, a very ſmall and highly-poliſhed iron wire, and in another obſerved obferved a manifeft alteration in the ftate of the mufcles, his opinion on this fubject was rendered lefs doubtful.

Frogs that he had deprived of their electricity, by means of the conductor, corrupted fooner than others which had not been deprived of it. In a note to this paffage the author afks, Am I not miftaken? Repeated difcharges, do they really deprive the animal of its natural electricity? or do they only put it in equilibrium? I know not, he fays, which of thefe to believe. It is certain, however, he adds, that frogs fatigued by the conductor, particularly in water, foon become putrid. What a curious difcovery, he obferves, it will be if it fhould hereafter be afcertained that the electric fluid retards the putrefaction of bodies. Before the prefent difcovery he knew, he tells us, that the fluid which circulates in the nerves is a powerful antifeptic.

Several frogs killed by the difcharge of the Leyden phial gave the fame figns of electricity as others that had not experienced fuch a fhock. The fuccefs of this experiment, however, the author obferves, muft depend on care being taken that the difcharge be not ftrong enough to diforganize the whole machine.

Frogs live ſeveral days in confined air without their electric quality ſeeming to ſuffer. Neither inflammable nor nitrous airs have been found to affect it; but it ſeemed, we are told, to experience a little diminution from phlogiſticated air; and it was much injured by air vitiated by the combuſtion of ſulphur. This effect, however, the author obſerves, was leſs perceptible in frogs that were only expoſed to it after being prepared, than in ſuch as had breathed and died in it. Under ſuch circumſtances the muſcular fibres were ſometimes found in a relaxed, and at others in a rigid and tenſe ſtate; and in the experiments the ſhocks were very weak, and after a few ſeconds could not by any means be excited. Is it a portion of electricity, the author aſks, which in this caſe is diſſipated? or is it the fibre which has loſt its natural ſtrength?

He has found that inflammable air extinguiſhes the life of a linnet or a Canary bird, but not their electricity, although it is naturally very weak.

He killed two kittens in phlogiſticated air; and having prepared their fore legs, found the uſual ſigns of electricity.

A dog

A dog was made to ſwallow arſenic, and died, but the poiſon was not found to have weakened his electricity. In other experiments hemlock was employed, and the reſult was the ſame.

If experiments ſhould ſhow that poiſons do not leſſen the electricity of animals, or, more properly, the capacity of parts to contain it, it will be neceſſary, our author thinks, to inquire why poiſoned animals corrupt more ſpeedily than others? It muſt be another principle of life, he obſerves, that has been offended. But where, he aſks, does it exiſt? Probably in the nerves, ſince miaſmata and venomous ſubſtances exert on them their firſt action. But our data on this ſubject are, he acknowledges, as yet too few to reaſon on.

Some frogs expoſed to the exhalation of putrid fleſh retained, after their death, weak ſigns of electricity.

Frogs killed by being placed under the exhauſted receiver of an air pump, and afterwards ſubjected to the uſual experiment, exhibited only feeble contractions, and theſe were excited with difficulty. In all of them an extravaſation of blood had taken place in the cellular membrane of the muſcles, ſo as to render their fleſh of a lively red colour. The blood being a conductor tor of electricity, it in this cafe, the author obferves, had difperfed a portion of it at the expence of the nerves, which are the road the fluid takes to reach the mufcles. On repeating the experiment with prepared frogs no fuch effufion was produced, and the electricity fhowed itfelf pretty well. — For thefe two experiments the author acknowledges himfelf indebted to Mr. Mofcati.

To a friend who had undertaken to explain, by means of the Leyden phial, all the phenomena of animal electricity, our author, after pointing out the difficulties of fuch an hypothefis, offered his own theory on the fubject, which is as follows:

The electric fluid, fays he, is either fent from the fenforium commune to the mufcles through the nerves, or returns to the fenforium through the infinite ramifications of thefe fame nerves from the whole furface of the body, or is diffufed through the body according to certain laws. In a word, electricity acts in the body in the manner phyfiologifts have fuppofed the nervous fluid to do.

To confirm this theory he contrived feveral experiments; among which the following, he thinks, appears of fome weight:

He

He took a frog, and, after removing its integuments, laid bare the vertebral column, which he divided above the origin of the crural nerves, and alſo at the origin of the lower extremities. The animal was thus divided into two parts, which communicated with each other only by the crural nerves. He coated theſe nerves, and placing one of the branches of the conductor on the coating, and the other on the trunk, the lower extremities were at that inſtant agitated as well as the upper parts and the fore feet.

If the experiment be repeated by tying the nerve, there is, he obſerves, no movement in the lower extremities.

If inſtead of placing the conductor on the trunk, it be placed on the ovaries, liver, lungs, head, or feet, the phenomenon, we are told, takes place equally. In this caſe, ſays the author, we do not eſtabliſh a communication between the external and internal ſurfaces of the muſcles, which are below the coating, and which notwithſtanding exhibit movements: it is the electric ſtream that paſſes from above downwards. Profeſſor Galvani himſelf, it ſeems, had remarked, that on making the experiment in a contrary direction the electricity of the lower extremities moved upwards; conſe-

 quently, quently, obſerves our author, the electric fluid circulates between the nervous filaments in all ſorts of directions. This, he thinks, is much in favour of his theory.

It is eaſy, he remarks, to prove that, without increaſing the degree of electricity, we may increaſe its celerity, as appears from the following experiment:—He took a frog, prepared as uſual, and directed againſt it a ſtream of electricity by means of a chain that communicated with its nerves. The animal, which at firſt was agitated, became after ſome little time immoveable. When it was in this ſtate, upon removing the conductor a little the frog renewed its motion, but ſoon fell into its former ſtate of inactivity. The current of electricity was then accelerated by applying an inſulated conductor to the muſcles of the frog, and the animal immediately began to move again. When it again ceaſed to move, by communicating with the conductor, he was again able to excite movements.

This ſhows, he thinks, that electricity is always the ſame, and that we only vary the manner of applying it.

We are not to ſuppoſe, however, he obſerves, that the ſame thing happens preciſely to the animal in full life. But he is convinced that

 there there exiſt in the animal cauſes capable of retarding or accelerating the electric current. He thinks we muſt ſeek for theſe cauſes in the different modes of ſenſation to be obſerved in the nerves; in the different properties of their cortical and medullary ſubſtances; and perhaps alſo in another principle, which exiſts in the nerves along with the electric fluid, and which is more or leſs combined with it. The ſubject, he is aware, is full of obſcurity. We ſhall, he obſerves, perhaps never ſee it clearly diſplayed, or, if we do, it will not be till after long and immenſe reſearches, and probably not till after we have been indulging in many erroneous ideas concerning it.

One great ſtep, however, he contends, is already made. The exiſtence of electricity has been demonſtrated in the animal machine; and this important diſcovery, he thinks, will tend to the explanation of many phenomena, among which he contents himſelf with mentioning the following:

Man and animals live a long time without refreſhing the blood with freſh chyle. If the blood were the fund which ſhould furniſh the principle that animates all the parts, and without which no movement or function of the animal mal œconomy could be executed, ſo great a waſte of life could not be of long duration. But now, he thinks, the myſtery is unfolded. The animal who takes no food attracts from the earth, and from the atmoſphere, this precious and neceſſary principle, the electric fluid.

It having been ſuggeſted to our author, that, in order to determine whether the nervous fluid be really the electric fluid or not, he ſhould have recourſe to the electrometer; and not happening at that time to be poſſeſſed of one ſufficiently ſenſible, he made the following experiment:

He prepared ſeveral frogs, the crural nerves of all which he united in a ſingle coating. Having put in order this battery, and eſtabliſhed a communication, by means of a conductor, between the nerves and the muſcles, he excited the electricity, and conſequently the movements. At the moment of the diſcharge two very ſmall bits of ſtraw, at a little diſtance the one from the other, but almoſt touching the apparatus, immediately approached each other. This experiment, he obſerves, proves the ſame thing as the electrometer would have done; but he has ſince, it ſeems, (as we are told in a note) em-

 ployed ployed the electrometer, and found that it gave figns of electricity.

On the day he wrote this account he, for the firft time, he tells us, coated the mufcles inftead of the nerves, and in this way obtained ftrong movements; but of this he propofes to fpeak more fully hereafter.

